# The relationship between bullying and stuttering in schoolchildren: scoping review

**DOI:** 10.1590/2317-1782/e20250428en

**Published:** 2026-08-07

**Authors:** Gislaine Aparecida Folha, Gabrielly Lemos Barbosa, Caio Rodrigues Felix, Hadassa Bighi Lima Baptista

**Affiliations:** 1 Faculdade de Medicina de Ribeirão Preto – FMRP, Universidade de São Paulo – USP - Ribeirão Preto (SP), Brasil.; 2 Núcleo de Apoio à Pesquisa em Morfofisiologia do Complexo Craniofacial da USP Universidade de São Paulo – USP - Ribeirão Preto (SP), Brasil.

**Keywords:** Stuttering, Childhood-Onset Fluency Disorder, Bullying, Speech Disorders, Child

## Abstract

**Purpose:**

to map and synthesise scientific evidence on the relationship between bullying and stuttering in schoolchildren. The following specific aims will be investigated: (1) the impact of the co-occurrence of both; (2) strategies for identifying bullying in children who stutter; and (3) approaches to tackling bullying in this population.

**Research strategies:**

Scoping Review was conducted following the Joanna Briggs Institute methodology. The PubMed, Scielo, CAPES, and Web of Science databases were consulted using specific descriptors.

**Selection criteria:**

Full-text articles in English or Portuguese were included, with no time limit. Opinion articles, letters to the editor, and studies that did not meet the objective of the study were excluded.

**Data analysis:**

The data extracted included author, title, objectives, methodology, results, and conclusions, culminating in a PRISMA-ScR flowchart for organising the findings.

**Results:**

The studies analysed point to a higher prevalence of bullying, anxiety and low self-esteem among children and adolescents who stutter, especially in the school environment. Weakened social relationships and lack of awareness increase the risk of exclusion. Speech therapists play an essential role in guidance and prevention, although there is still a need for more robust research on effective interventions.

**Conclusion:**

This study confirmed the relationship between stuttering and the experience of bullying, highlighting the impacts on the mental health and socialisation of people who stutter. In addition, although scarce, the main intervention strategies identified to date were gathered, such as educational programmes and prevention activities, still in the school environment and in conjunction with the academic staff and family members.

## INTRODUCTION 

Although there are several definitions in the literature, bullying is understood as repetitive acts carried out by an individual or group with the intention of harming an individual or group that shares common characteristics; therefore, it should be considered unacceptable and inappropriate^(1)^. Bullying occurrences may manifest in physical, verbal, or social forms, or through interpersonal interactions such as social isolation^(1)^. These behaviors may occur in various contexts, including school, family environments, virtual interactions (cyberbullying), or among peers, involving a power imbalance between the perpetrator and the victim, who feels inferior^(1)^.

Bullying is considered a global epidemic among schoolchildren, with rates ranging from 49% to 58% among elementary school students, and may represent a significant problem for children who stutter^(1)^, as they experience bullying more frequently compared to peers who do not stutter^(2)^. Among children who stutter, the incidence of bullying reaches up to 81%^(3)^.

Furthermore, bullying behavior among children who stutter is frequently associated with name-calling, defamation, feelings of guilt, physical aggression, and ridicule^(4)^. In the school environment, this may reduce self-esteem, impair academic performance, increase social rejection, and lead to depression, feelings of helplessness, and loneliness. For children with speech fluency disorders, bullying may exacerbate stuttering, intensify negative emotions, and reduce therapeutic progress^(5)^. The consequences of bullying may be long-lasting, extending into adulthood, resulting in higher levels of social anxiety, fear of negative evaluation, and reduced quality of life^(6,7)^.

In children who stutter, bullying may occur because their disfluencies are difficult for others to understand, who may not comprehend why their speech differs from what society considers “standard.” This highlights the importance of educating peers, family members, and educators about stuttering so they can respond constructively in situations of intimidation^(1)^, especially for children who often do not yet know how to respond to such aggression.

Speech-language pathologists, in partnership with teachers, should lead efforts to help children who stutter cope with bullying. Empowering these children may be an effective strategy to combat bullying. However, a lack of understanding regarding the role of speech-language pathologists in this aspect of treatment, or uncertainty about how to address bullying, may hinder this practice^(8)^. Although speech-language pathologists are a reference for these complaints, they are not always prepared to deal with this issue^(1)^.

In order to organize information and highlight the contribution of speech-language pathologists in this area, it is necessary to promote a better understanding of how bullying occurs among schoolchildren, the impact of bullying on Childhood-Onset Fluency Disorder (stuttering), and to map and synthesize scientific evidence regarding the relationship between bullying and stuttering.

It should be noted that a preliminary search did not identify any literature review studies on this topic, highlighting the importance of the present study for current contextualization of the issue and for identifying recommendations for future research focused on the correlation between the impact of Childhood-Onset Fluency Disorder and bullying. Thus, there is a need to fill gaps in the scientific literature in order to advance strategies to address this issue.

Thus, the general objective of this study was to map and synthesize scientific evidence on the relationship between bullying and stuttering in schoolchildren, with specific objectives of describing the impact of their co-occurrence, identification strategies, and possible coping strategies.

## METHODS

### Research strategy

The literature search was conducted through the databases PubMed, Scielo, CAPES, and Web of Science. These databases were selected due to their methodological criteria, including thematic coverage, indexing quality, and relevance. The review included publications up to June 2025, with no prior time restriction.

The descriptors used were “Bullying,” “Stuttering,” “Stutter,” “Tachyphemia,” “Fluency disorder,” and “Neurodevelopmental speech fluency disorder,” as well as their Portuguese equivalents.

Three reviewers (GLB, CRF, and HBLB) independently screened titles and abstracts to determine eligibility. Reviewer training was conducted by the lead researcher to ensure consistency in selection and data extraction. Each reviewer populated an Excel spreadsheet (Microsoft® Corporation) with titles, keywords, and abstracts, indicating inclusion or exclusion (Chart 1).

**Chart 1:** Electronic databases and search strategies

**Electronic database**	**Strategy (descriptors)**
Pubmed	(Bullying AND Gagueira), (Bullying AND stuttering OR stutter), (Bullying AND Taquifemia), (Bullying AND Tachyphemia), (Bullying AND Distúrbios da fluência da fala), (Bullying AND Fluency disorder), (Bullying AND Distúrbios do neurodesenvolvimento da fluência da fala), (Bullying AND Neurodevelopmental speech fluency disorder)
Scielo	(Bullying AND Gagueira), (Bullying AND stuttering OR stutter), (Bullying AND Taquifemia), (Bullying AND Tachyphemia), (Bullying AND Distúrbios da fluência da fala), (Bullying AND Fluency disorder), (Bullying AND Distúrbios do neurodesenvolvimento da fluência da fala), (Bullying AND Neurodevelopmental speech fluency disorder)
CAPES	(Bullying AND Gagueira), (Bullying AND stuttering OR stutter), (Bullying AND Taquifemia), (Bullying AND Tachyphemia), (Bullying AND Distúrbios da fluência da fala), (Bullying AND Fluency disorder), (Bullying AND Distúrbios do neurodesenvolvimento da fluência da fala), (Bullying AND Neurodevelopmental speech fluency disorder)
Web of Science	(Bullying AND Gagueira), (Bullying AND stuttering OR stutter), (Bullying AND Taquifemia), (Bullying AND Tachyphemia), (Bullying AND Distúrbios da fluência da fala), (Bullying AND Fluency disorder), (Bullying AND Distúrbios do neurodesenvolvimento da fluência da fala), (Bullying AND Neurodevelopmental speech fluency disorder)
Google Scholar	(Bullying AND Gagueira), (Bullying AND stuttering OR stutter), (Bullying AND Taquifemia), (Bullying AND Tachyphemia), (Bullying AND Distúrbios da fluência da fala), (Bullying AND Fluency disorder), (Bullying AND Distúrbios do neurodesenvolvimento da fluência da fala), (Bullying AND Neurodevelopmental speech fluency disorder)

Following this stage, consensus meetings were held to determine study inclusion. In cases of disagreement, a third reviewer was consulted.

### Eligibility criteria

Studies included were full-text articles in English or Portuguese addressing the defined descriptors. Opinion articles, letters to the editor, and studies not aligned with the study objective were excluded. No publication time limits were established.

Only studies meeting all eligibility criteria were included in the analysis.

### Data analysis

At this stage, evaluators included a speech-language pathologist with a doctoral degree and an undergraduate student in Speech-Language Pathology to ensure conceptual alignment. Studies were read in full and independently assessed to include only those addressing the impact of co-occurring bullying and stuttering, identification strategies, and/or coping strategies.

Data were recorded in a structured spreadsheet and organized using Mendeley. Extracted variables included author, title, year, language, objectives, study type, methodology, results, and conclusions.

**Figure 1:**
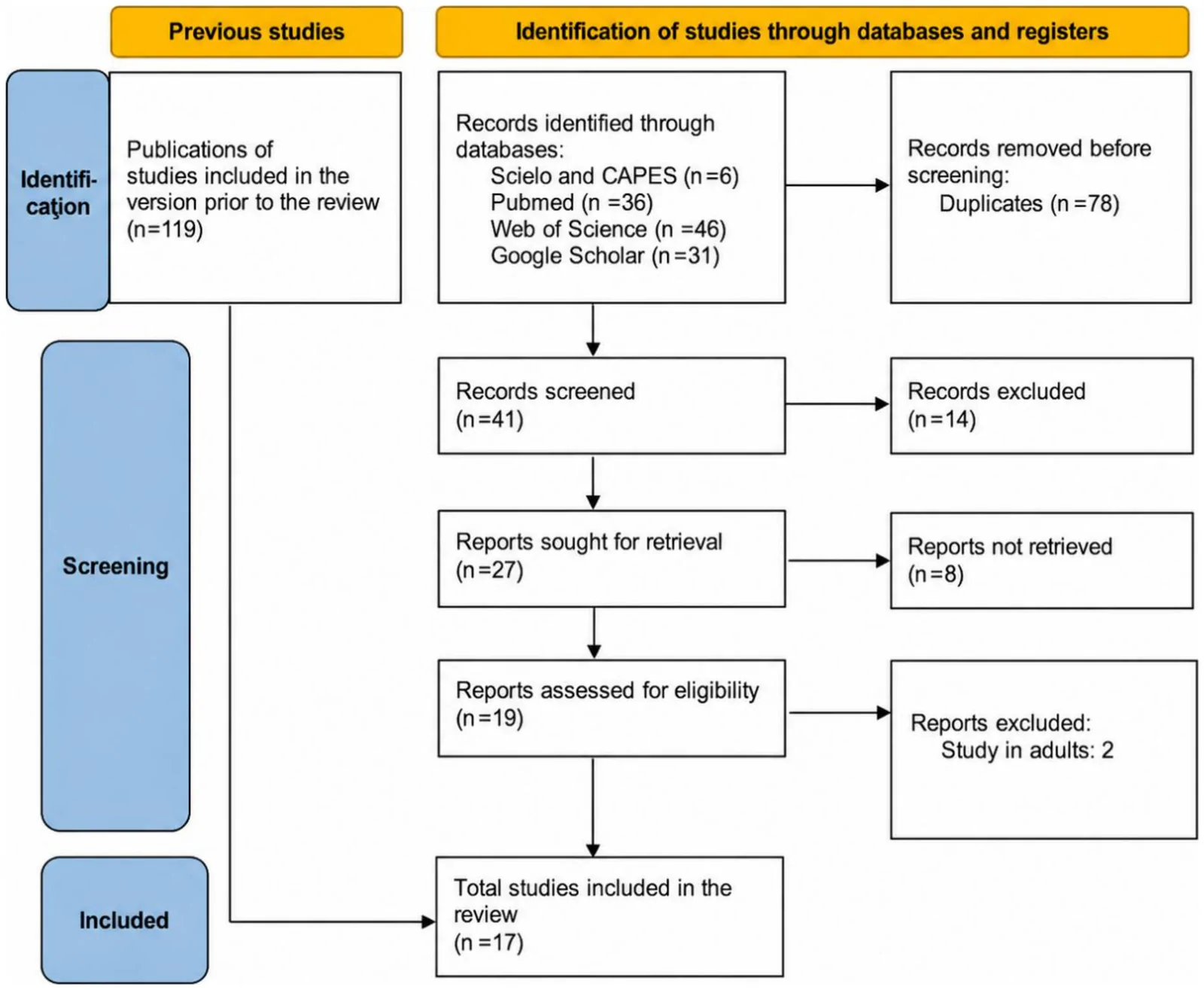
Flowchart of the study selection process

The selection process is summarized in the PRISMA-ScR flowchart (Figure 1).

This review followed the Joanna Briggs Institute (JBI) scoping review methodology^(9)^, based on the PCC framework (Population, Concept, Context). The guiding question was: “What is the relationship between bullying and stuttering in school-age children?” with P: schoolchildren; C: stuttering and bullying; C: school context.

The study strictly followed the PRISMA-ScR checklist (Appendix 1).

## RESULTS

### Results from electronic databases

The initial search identified 119 articles. After removing duplicates, 41 articles were screened. Of these, 27 were selected for full-text reading. Ultimately, 17 studies met the eligibility criteria and were included.

The selection process is illustrated in Figure 1 and in Chart 2.

**Chart 2:** Presentation of studies that met the eligibility criteria

**Authors**	**Title**	**Year**	**Language**	**Objectives**	**Type of study**	**Methodology**	**Results**	**Conclusions**
Mallick R, Kathard H, Borhan ASM, Pillay M, Thabane L	A cluster randomised trial of a classroom communication resource program to change peer attitudes towards children who stutter among grade 7 students	2018	English	The primary objective of this study was to determine the treatment effect six months after the intervention among 7th-grade participants (intervention with Classroom Communication Resources [CCR] versus no CCR) using overall scores from the Stuttering Resource Outcomes Measure (SROM) in school groups. The secondary objective was to determine the treatment effect among 7th-grade participants on the SROM subscales, including Positive Social Distance (PSD), Social Pressure (SP), and Verbal Interaction (VI). The subgroup objective was to determine any differences in the primary outcome between schools across quintile groups (lower and higher).	Experimental	After schools were stratified into lower and upper quintile subgroups (defined according to geographic location, school fees, and resources), they were randomly assigned to control and intervention groups, comprising 7th-grade participants generally aged 11 years or older. Teachers received one hour of training prior to administering the single-dose CCR intervention during a 60 to 90-minute session. The CCR intervention included a social story, role-playing, and discussion. All participants watched a video of a child who stutters (CWS), and stuttering was defined at baseline. The SROM measured peer attitudes six months after the intervention. Randomization was stratified by quintile group using a 1:1 allocation ratio. A fully blinded trial was not feasible; however, the outcome assessor was partially blinded and the analyst was blinded. Generalized estimating equations (GEE), assuming an exchangeable correlation structure, were used to analyze the data under an intention-to-treat principle. Multiple imputation was used to handle missing data. Statistical significance was set at α = 0.05.	Ten schools were randomly allocated to the control (k = 5) and intervention (k = 5) groups, with n = 223 participants in the intervention group and n = 231 in the control group. A total of 454 participants completed the SROM in the control (n = 231) and intervention (n = 223) groups and were analyzed at baseline and six months post-intervention. There was no statistically significant difference in the overall SROM score (mean difference −0.11; 95% confidence interval [CI] −1.56 to 1.34; p = 0.88). There were also no significant differences in the SROM subscales: PSD (mean difference 1.04; 95% CI −1.02 to 3.11; p = 0.32), SP (mean difference −0.45; 95% CI −1.22 to 0.26; p = 0.21), and VI (mean difference 0.05; 95% CI −1.01 to 1.11; p = 0.93). In addition, there was no significant subgroup effect on the overall SROM score (lower versus upper quintile subgroups) (interaction p-value = 0.52). No harms were observed or reported.	No statistically significant differences were observed. It is possible that the time period was too short to detect changes in peers’ attitudes and that further studies are needed to confirm these findings.
Mallick RB, Thabane L, Borhan ASM, Kathard H	A pilot study to determine the feasibility of a cluster randomised controlled trial of an intervention to change peer attitudes towards children who stutter	2018	English	This pilot study therefore aimed to determine the feasibility of a randomized controlled trial (RCT) by exploring: (1) procedural issues and (2) the treatment effect of the Classroom Communication Resource (CCR), an intervention designed to change peers’ attitudes toward children who stutter.	experimental	A stratified cluster randomized controlled trial (RCT) pilot design was employed, in which recruitment first occurred at the school level and subsequently at the individual level. Attrition rates were reported at baseline, and at 1 and 6 months post-intervention. For the treatment effect, schools were the unit of randomization and were assigned to receive either the teacher-delivered CCR intervention or usual practice, using a 1:1 allocation ratio. The Stuttering Resource Outcomes Measure (SROM) was used to assess treatment effects at baseline, and at 1 and 6 months following the intervention, both overall and across its constructs (positive social distance, social pressure, and verbal interaction).	For school recruitment, 11 schools were invited to participate and 82% (n = 9) were recruited. Based on school recruitment, N = 610 participants were eligible for the study, whereas only n = 449 were recruited, with n = 183 in the intervention group and n = 266 in the control group. The attrition rate from recruitment to baseline was as follows: intervention, 23% (n = 34), and control, 6% (n = 15). At 1 month, an attrition rate of 7% (n = 10) was observed in the intervention group and 6% (n = 15) in the control group, while at 6 months, attrition rates of 7% (n = 10) and 17% (n = 44) were observed in the intervention and control groups, respectively. Regarding the treatment effect on the SROM, the estimated mean differences between the intervention and control groups were (95% confidence interval [CI]: −1.07, 5.11) at 1 month and 3.01 (95% CI: −0.69, 6.69) at 6 months. A statistically significant difference was observed at 6 months in the SROM VI subscale, with a value of 1.35 (95% CI: 0.58, 2.13).	A high recruitment rate of schools and participants was observed, along with a high participant attrition rate. Significant differences were observed only at 6 months post-intervention within one of the SROM constructs. These findings suggest that a future randomized controlled trial (RCT) is warranted and feasible.
Mandrá PP, Cassini M, Gonçalves TC, Kuroishi RCS	Educational health actions: satisfaction regarding the stuttering awareness campaign	2017	English	To investigate satisfaction with the health education actions carried out during the Stuttering Awareness Campaign.	Cross-sectional observational study	The sample consisted of archived institutional documents referring to 135 questionnaires administered and fully completed during the Stuttering Awareness Campaign. These were structured questionnaires composed of closed-ended questions, including Likert-scale responses to assess satisfaction, two dichotomous questions regarding bullying (“yes”/“no”), and one open-ended question addressing “what was lacking in the campaign.” The data obtained were categorized and tabulated for analysis using nonparametric statistics. Absolute and relative frequencies of responses were calculated. For analysis, participants were divided into two groups according to the campaign setting: G1 (specialized service, n = 31) and G2 (schools, n = 104).	Educational actions were predominantly carried out through lectures (89.6%) and group discussions (10.4%). All participants had access to the informational leaflet and T-shirt; 84.4% had access to the video and poster. Most participants reported satisfaction with the topic and duration of the campaign. The explanations were considered sufficient to understand what bullying is (97.9%); 57.6% reported knowing someone who had experienced some form of intimidation (6.0% related to stuttering). Regarding what was lacking in the campaign, a diversity of responses was observed, with the highest percentage referring to “forms of treatment” (11.9%), followed by “physical aggression in schools” (0.9%).	There was satisfaction with the health education actions conducted during the 2010 Stuttering Awareness Campaign regarding the topic, duration, type of activities, and materials accessed.
Blood GW, Boyle MP, Blood IM, Nalesnik GR	Bullying in children who stutter: speech-language pathologists’ perceptions and intervention strategies	2010	English	The present study had three main objectives: (1) to determine the perceived severity and likelihood of intervention in episodes of physical, verbal, and relational bullying involving students who stutter, as evaluated by school-based speech-language pathologists; (2) to explore speech-language pathologists’ perceptions of potential strategies/interventions they considered appropriate, based on six different vignettes describing physical, verbal, and relational bullying of students who stutter; and (3) to determine any associations between speech-language pathologists’ responses and (a) their age, (b) years of professional experience, (c) the number of children who stutter currently on their caseload, and (d) caseload size, as these factors may be related to their perceptions and ratings of specific intervention strategies.	Longitudinal observational study	For this study, a proportional, stratified, and probabilistic random sampling technique was used (Dillman et al., 2008; Fink, 2008). The sample was proportional to the number of speech-language pathologists (SLPs) in each state and stratified across all 50 states. A mail survey methodology was employed because it allowed for a broader range of potential participants across multiple locations in the United States. Additionally, it is a preferred method for studying sensitive topics in a discreet, confidential, and anonymous manner (Babbie, 2006; Babbie, 2007; Dillman et al., 2008; Fink, 2008).	The main findings of this study were that: (1) both perceived severity and likelihood of intervention were highest for physical bullying, followed by verbal bullying, with relational bullying consistently rated as the lowest by school-based speech-language pathologists; (2) references to stuttering in bullying scenarios were generally not perceived as more serious or more likely to require intervention than scenarios in which stuttering was not referenced. Furthermore, the inclusion of stuttering led to significant differences in ratings for only two of the nine intervention strategies; and (3) perceived severity, likelihood of intervention, and use of intervention strategies did not differ according to demographic factors or work practices.	The conclusions of the study highlight the need for a more systematic approach to addressing bullying in children who stutter within speech-language pathology practice. Speech-language pathologists recognize the importance of integrating intervention strategies that not only focus on speech fluency but also address the social and emotional aspects of stuttering..
Kira CF, Alpes MF, Mandrá PP	Construction and content validation of educational material on stuttering	2023	English	To develop and validate the content of illustrative material to guide families and educators of children who stutter	Revision	Methodological study following the steps recommended in the literature for the validation of educational materials. Three groups of judges were selected: eight speech-language pathologists, ten early childhood educators, and ten family members of children and adolescents who stutter. The judges completed a self-administered questionnaire to evaluate the content, language, illustrations, layout, and motivational aspects of the materials. After reading the booklet, they were asked to indicate their level of agreement using a Likert scale: (1) strongly disagree, (2) disagree, (3) neither agree nor disagree, (4) agree, (5) strongly agree, followed by three dichotomous questions (yes/no) with space provided for comments and suggestions.	Individual suggestions and comments from the judges regarding content and language were considered. The final version was produced on an A4 sheet, landscape format, with three panels, organized as follows: front cover; back cover including incidence and types (developmental, neurogenic, and psychogenic); diagnosis and treatment; favorable attitudes; and a note on bullying in stuttering, in addition to the definition and main manifestations of stuttering.	The material was developed and its content validated, demonstrating a high level of acceptance and agreement among the judges, serving as a resource for healthcare and education professionals, as well as for parents, regarding the topic of stuttering.
Lowe R, Jelčić Jakšić S, Onslow M, O’Brian S, Vanryckeghem M, Millard S, et al.	Contemporary issues with stuttering: The Fourth Croatia Stuttering Symposium	2021	English	To document and discuss current clinical issues related to stuttering as addressed during the symposium.	Revision	It consists of documenting the discussions that took place during the symposium held in 2019. The authors prepared a written record that faithfully reproduces the topics addressed, aiming to identify the main contemporary clinical issues related to stuttering. The approach included collecting insights from speech-language pathologists, academics, and researchers from 29 countries, who shared their experiences and perspectives on the management of stuttering.	They indicate that early intervention is crucial for the effective management of stuttering; however, there is still no consensus on the best approach for its implementation. The study also highlights the difficulty in translating evidence-based practices into clinical care and notes the anxiety that children may experience during treatment. Additionally, the authors recommend conducting appropriate assessments for persistent stuttering and suggest that speech-language pathologists are the ideal professionals to provide cognitive-behavioral therapy to these patients.	They emphasize the importance of early intervention for the effective treatment of stuttering, although there is still no consensus regarding the timing and management of these interventions.
Murphy WP, Yaruss JS, Quesal RW	Enhancing treatment for school-age children who stutter II. Reducing bullying through role-playing and self-disclosure	2007	English	To investigate ways to reduce bullying experienced by school-age children who stutter using role-playing and self-disclosure techniques. The study aims to evaluate how these approaches can improve children’s communication, increase their confidence, and help them cope effectively with bullying in the school environment.	Experimental study	School-age children who stutter participate in intervention sessions using role-playing and self-disclosure techniques. During the sessions, children practice different social scenarios, simulating bullying situations, to learn how to respond effectively. Self-disclosure is also encouraged, helping children speak openly about their stuttering. The impact of these interventions is evaluated based on changes in children’s behavior and reductions in reported bullying.	The study results showed that role-playing and self-disclosure techniques helped reduce bullying among children who stutter. Participants reported increased confidence when dealing with difficult social situations and demonstrated improvements in how they addressed bullying. There was also a significant reduction in reported bullying occurrences following the implementation of these techniques, indicating that these strategies were effective in promoting more assertive responses and improving children’s social interactions.	Role-playing and self-disclosure techniques are effective in reducing bullying experienced by children who stutter. The study demonstrates that these interventions increase children’s confidence, improve their communication skills, and help them cope with bullying situations in a more assertive manner.
Kikuchi Y, Umezaki T, Sawatsubashi M, Taura M, Yamaguchi Y, Murakami D, Nakagawa T	Experiences of Teasing and Bullying in Children Who Stutter	2019	English	To investigate the frequency and impact of teasing and bullying experiences among children who stutter, particularly considering age and the type of experience reported.	Cross-sectional observational study	The sample comprised 120 children who stutter (CWS), aged between 3 and 12 years, treated at a university hospital in Japan, where they were individually interviewed.	66.6% of the children reported at least one negative experience related to their speech; reported experiences increased with age, particularly from 5 years onward; most children who experienced these situations reported negative feelings, such as sadness and discomfort.	Many children who stutter are targets of questions, imitation, and laughter from others, even at very early ages. These situations are perceived as uncomfortable or distressing by most children. The authors recommend that healthcare professionals ask children directly about these experiences and intervene early, promoting the prevention of bullying and protecting the emotional health of these children.
Zamprogno MP, Mandrá PP, Gonçalves TC, Jorge TM	Experiences of school violence from the perspective of patients who stutter	2020	English	To characterize school-age patients with persistent stuttering regarding their self-reported experiences of violence in the school context.	Cross-sectional observational study	The sample consisted of 10 patients with persistent stuttering receiving care at a fluency outpatient clinic in the city of Ribeirão Preto, São Paulo, Brazil, aged between 10 and 17 years, regardless of sex and stuttering characteristics. The data collection instrument was a self-administered questionnaire containing 11 multiple-choice questions. Data were analyzed descriptively by presenting the frequency of responses.	Nearly half of the patients who stutter reported experiencing bullying, including name-calling, being blamed for various situations, defamation, physical aggression, and mockery. The classroom was the most frequently reported setting for bullying occurrences. Reported reactions to the experienced violence included “talking to friends, teachers/principals, and family members,” as well as “sadness” and a “desire to change schools.”	Despite the small sample size, alarming findings were observed, highlighting the importance of educational and preventive actions in the school environment regarding both bullying and stuttering.
Bernard RFL, Norbury CF.	Factors Associated With Symptoms of Anxiety and Depression in Children Who Stutter	2023	English	This study examines the association between symptoms of anxiety and depression and stuttering, as well as child, family, and contextual factors that may influence this association.	Cross-sectional observational study	Thirty-five school-age children who stutter completed the Revised Children’s Anxiety and Depression Scale–Short Version. Regression models were fitted to examine the association between symptoms of anxiety and depression and bullying, stuttering severity, adverse family mental health history, and age in children who stutter.	Adverse family mental health history was found to significantly predict anxiety and depression scores. Age also predicted depression scores, with older children reporting higher scores.	Adverse family mental health history is associated with higher self-reported internalizing symptoms in children who stutter. The interaction among child, family, and contextual factors may change with age, warranting further investigation in larger longitudinal studies. The association between bullying and anxiety scores highlights the importance of anti-bullying initiatives in promoting psychosocial development in school-age children who stutter. This study also underscores the contribution of known mental health risk factors, such as family history, to variability in reported symptoms.
Silva LK, Martins-Reis V de O, Maciel TM, Ribeiro JKBC, Souza MA de, Chaves FG	Stuttering at school: effect of a teacher training program on stuttering	2016	English	To assess what teachers from public and private schools know about stuttering, as well as to evaluate the effectiveness of the Teacher Training Program on Stuttering in expanding this knowledge.	Cross-sectional observational study	A total of 137 early childhood education teachers participated in the study. Initially, the teachers completed a questionnaire about stuttering. Subsequently, 75 teachers participated in the Teacher Training Program on Stuttering, which lasted four hours. One month after participating in the program, the teachers completed the questionnaire again.	After the training, there was an increase in the proportion of participants who considered the prevalence of people who stutter in the population to be low. Respondents began to report that stuttering is more frequent in males. There was an increase in those who considered stuttering to be hereditary. The proportion of educators who believed that stuttering is psychological decreased. Most respondents came to believe in the multifactorial nature of stuttering. The proportion of educators who stated that stuttering is emotional decreased. There was improved understanding of certain attitudes that educators can adopt to help individuals who stutter.	Educators demonstrated some knowledge about stuttering; however, it was insufficient to differentiate it from other language disorders. The program increased knowledge about stuttering. However, it proved to be more effective in improving understanding of stuttering characteristics than in changing educators’ attitudes.
Davis S, Howell P, Cooke F.	Sociodynamic relationships between children who stutter and their non-stuttering classmates	2002	English	To investigate social interactions between children who stutter and their non-stuttering peers. The study aims to understand the dynamics of acceptance, rejection, and social isolation that may occur between these groups, as well as the impact of stuttering on friendships, group behavior, and peers’ social perceptions.	Cross-sectional observational study	The study methodology consisted of an observational analysis of social interactions between children who stutter and their peers. Researchers used questionnaires and direct observations in the school setting to assess social behaviors, levels of acceptance and rejection, and friendship networks among participants. The focus was to identify interaction patterns and understand how stuttering influences relationships within the group.	The study results indicate that children who stutter experience higher levels of social isolation and rejection from their non-stuttering peers. Their social interactions are more limited, and they tend to have fewer friends. However, some peers demonstrate support and acceptance, which varies according to the school environment and group dynamics. The study highlights the importance of social interventions to improve the inclusion of these children.	Children who stutter face significant social challenges in their interactions with peers, such as exclusion and difficulties in forming friendships. The study suggests that these difficulties may impact their social and emotional development. The authors recommend that schools implement awareness programs and interventions that promote inclusion to improve the social experiences of children who stutter.
McAllister J, Skinner J, Hayhow R, Heron J, Wren Y.	The Association Between Atypical Speech Development and Adolescent Self-Harm	2023	English	To investigate the relationship between atypical speech development in children and self-harm in adolescence, as well as to explore how communication difficulties may affect adolescents’ mental health. The study also aims to highlight the importance of early interventions for children with these difficulties in order to prevent self-harming behaviors in the future.	Cross-sectional observational study	Through the analysis of a database, the researchers collected information on demographic characteristics and risk factors, using standardized scales to assess the relationship between speech development and self-harm behaviors.	The study found a significant association between atypical speech development in children and an increased risk of self-harm in adolescence. The results indicate that children with communication difficulties are more likely to develop emotional and behavioral problems, including self-injurious behaviors. The authors emphasize the need for early interventions to support children with atypical speech development in order to mitigate these risks during adolescence.	The study concludes that atypical speech development in children is associated with an increased risk of self-harm in adolescence.
Kara I, Mutlu Aİ, Miraloğlu K	Comparison of Participation in Online Games and Communication Experiences of School-Age Children Who Do and Do not Stutter: Exploratory Study	2023	English	To investigate whether there are significant differences in participation in online games and in communication experiences between children who stutter (CWS) and those who do not stutter (CWNS). The study also aims to understand whether these interactions in the gaming environment may influence speech characteristics and the occurrence of behaviors such as bullying, providing important insights into how the digital environment affects the communication of children who stutter.	Cross-sectional observational study	Online questionnaires were administered to assess participation habits in multiplayer games, frequency of use, and speech characteristics during gameplay. The study involved 91 children who stutter and 116 who do not, allowing for comparisons in communication experiences and the occurrence of bullying behaviors during gaming.	The findings showed that children who stutter and those who do not participate in online games in a similar manner, with no significant differences in frequency or duration. However, children who stutter reported fewer occurrences of bullying behaviors during gaming, indicating that the online environment may be safer and more favorable for them.	Online gaming environments may offer positive opportunities for communication and a safer space for children who stutter
Karahan Tığrak T, Kulak Kayıkcı ME, Kirazlı MÇ, Tığrak A	Emotional and behavioural problems of children and adolescents who stutter: Comparison with typically developing peers	2020	English	To investigate and compare emotional and behavioral problems in children and adolescents who stutter with those of their typically developing peers. The study aims to identify significant differences in levels of anxiety, depression, and other emotional and behavioral difficulties between the groups, as well as to better understand the impact of stuttering on the emotional well-being of children and adolescents.	Cross-sectional observational study	It consisted of comparing two groups: children and adolescents who stutter and typically developing children. Researchers used questionnaires to collect data on emotional and behavioral problems, applying standardized instruments such as the Strengths and Difficulties Questionnaire (SDQ), which assesses aspects such as hyperactivity and emotional and behavioral difficulties. The sample included children and adolescents of various ages, and the data were statistically analyzed to identify significant differences between the groups.	Children and adolescents who stutter present significantly higher levels of emotional and behavioral problems compared to their typically developing peers. The data showed that these individuals were more likely to report difficulties such as anxiety, depression, and hyperactivity-related behaviors. Specifically, the analyses revealed that participants who stutter had higher scores on the Strengths and Difficulties Questionnaire (SDQ) across several domains, suggesting a negative impact of stuttering on emotional well-being. These findings highlight the need for psychological support and targeted interventions for children who stutter to improve their mental and social health.	The authors emphasize the importance of recognizing these difficulties and suggest that health and education professionals should implement interventions that address both stuttering and the associated emotional issues.
Logan KJ, Mullins MS, Jones KM	The depiction of stuttering in contemporary juvenile fiction: Implications for clinical practice	2008	English	To analyze how stuttering is represented in contemporary juvenile literature and to discuss the implications of these representations for clinical practice in speech-language pathology. The authors sought to determine whether these books could be used as educational or therapeutic tools in the treatment of stuttering in children and adolescents.	Revision	To select and critically review 29 juvenile fiction books published after 1988 that featured child or adolescent characters who stutter. For selection, the authors conducted searches in libraries, bookstore websites, and academic databases, and consulted experts and librarians. The selected books were evaluated based on the accuracy of clinical representations of stuttering, depth of themes addressed, personal and social factors, and potential functional gains presented by the characters.	Most stories portray characters who initially face significant difficulties related to stuttering, such as isolation, bullying, and low self-esteem, and many show positive development over the course of the narrative, although not always associated with professional treatment. In general, the books address the emotional and social aspects of stuttering well, but they often present inaccurate or oversimplified explanations regarding the causes and treatment of the disorder, reinforcing the idea that it is linked to emotional trauma or can be overcome through emotional support and social acceptance.	The authors indicate that juvenile literature can be a valuable resource for use in speech-language pathology interventions, particularly in activities involving education, the promotion of empathy, demystification of the disorder, and encouragement of self-expression. Despite limitations, such as the limited mention of formal speech-language therapy and an excessive focus on emotional factors, the analyzed books demonstrate potential to support clinical practice, especially when used with professional guidance and supplemented with accurate information about stuttering.
Blood GW; Blood IM	Preliminary Study of Self-Reported Experience of Physical Aggression and Bullying of Boys Who Stutter: Relation to Increased Anxiety	2007	English	To investigate the relationship between experiences of physical aggression and bullying in boys who stutter, analyze the impact of these experiences on boys’ anxiety, and identify patterns of aggression and bullying reported by participants, seeking to understand how these factors affect their emotional and psychological health.	Cross-sectional observational study	Data were collected through self-administered questionnaires. Participants, who were boys who stutter, reported their experiences of physical aggression and bullying, as well as their levels of anxiety. Data analysis included comparing the boys’ responses regarding their bullying experiences and reported anxiety levels, aiming to identify significant relationships between these factors.	Boys who stutter reported significant experiences of physical aggression and bullying. These reports were associated with higher levels of anxiety among participants. The study suggests that stuttering may increase these boys’ vulnerability to bullying experiences, which in turn contributes to anxiety and other emotional problems.	Stuttering may increase boys’ exposure to physical aggression and bullying, resulting in elevated levels of anxiety. The authors emphasize the importance of recognizing these experiences and their emotional repercussions, suggesting that targeted interventions are necessary to provide support for boys who stutter.

## DISCUSSION

This study aimed to map and synthesize scientific evidence on the relationship between bullying and stuttering in schoolchildren, considering impact, identification strategies, and coping strategies.

Among the 17 included studies, most reported a high prevalence of bullying^(4,10-15)^, elevated anxiety levels^(10,15)^, and lower self-esteem^(10)^ among children who stutter compared to those who do not. Adolescents who stutter are at significantly higher risk of bullying^(12,16)^.

These findings support a significant association between bullying and stuttering, suggesting that childhood fluency disorder may act as both a vulnerability factor and a target for stigmatization in school contexts. The classroom was identified as the primary setting for bullying, emphasizing the urgency of preventive strategies^(4)^.

Children who stutter often experience peer rejection, lower social status, and difficulties forming friendships^(17)^. Teacher-student relationships and adult awareness were identified as factors influencing bullying risk^(18)^. Educational programs and school-based prevention strategies may improve peer attitudes^(17,19)^.

Speech-language pathologists play a crucial role in identifying and addressing bullying, providing guidance, promoting acceptance, and teaching assertive responses^(12,20)^. However, limitations include the predominance of cross-sectional designs, heterogeneous assessment tools, and incomplete data, affecting generalizability.

Although Fluency is recognized as a specialty in Brazil (CFFa Resolution nº 507/2017)^(21)^, only four studies in this review were conducted by Brazilian authors^(4,11,19,22)^.

## CONCLUSION

This study confirmed the relationship between stuttering and bullying, highlighting impacts on mental health and socialization. Although limited, intervention strategies identified include educational programs and preventive activities within school settings involving academic staff and families.

Future studies should evaluate the effectiveness of interventions and their application in clinical practice. There is a need for observational and experimental studies with larger samples to investigate therapeutic outcomes in real-life contexts.

## Appendix 1 Checklist PRISMA-ScR

Preferred Reporting Items for Systematic reviews and Meta-Analyses extension for Scoping Reviews (PRISMA-ScR) Checklist

**Table:**

**SECTION**	**ITEM**	**PRISMA-ScR CHECKLIST ITEM**	**REPORTED ON PAGE #**
**TITLE**			
Title	1	Identify the report as a scoping review.	1
**ABSTRACT**			
Structured summary	2	Provide a structured summary that includes (as applicable): background, objectives, eligibility criteria, sources of evidence, charting methods, results, and conclusions that relate to the review questions and objectives.	1
**INTRODUCTION**			
Rationale	3	Describe the rationale for the review in the context of what is already known. Explain why the review questions/objectives lend themselves to a scoping review approach.	5
Objectives	4	Provide an explicit statement of the questions and objectives being addressed with reference to their key elements (e.g., population or participants, concepts, and context) or other relevant key elements used to conceptualize the review questions and/or objectives.	6
**METHODS**			
Protocol and registration	5	Indicate whether a review protocol exists; state if and where it can be accessed (e.g., a Web address); and if available, provide registration information, including the registration number.	8
Eligibility criteria	6	Specify characteristics of the sources of evidence used as eligibility criteria (e.g., years considered, language, and publication status), and provide a rationale.	7
Information sources*	7	Describe all information sources in the search (e.g., databases with dates of coverage and contact with authors to identify additional sources), as well as the date the most recent search was executed.	6
Search	8	Present the full electronic search strategy for at least 1 database, including any limits used, such that it could be repeated.	6
Selection of sources of evidence†	9	State the process for selecting sources of evidence (i.e., screening and eligibility) included in the scoping review.	7
Data charting process‡	10	Describe the methods of charting data from the included sources of evidence (e.g., calibrated forms or forms that have been tested by the team before their use, and whether data charting was done independently or in duplicate) and any processes for obtaining and confirming data from investigators.	8
Data items	11	List and define all variables for which data were sought and any assumptions and simplifications made.	7
Critical appraisal of individual sources of evidence§	12	If done, provide a rationale for conducting a critical appraisal of included sources of evidence; describe the methods used and how this information was used in any data synthesis (if appropriate).	6
Synthesis of results	13	Describe the methods of handling and summarizing the data that were charted.	9
**RESULTS**			
Selection of sources of evidence	14	Give numbers of sources of evidence screened, assessed for eligibility, and included in the review, with reasons for exclusions at each stage, ideally using a flow diagram.	8 and 38
Characteristics of sources of evidence	15	For each source of evidence, present characteristics for which data were charted and provide the citations.	19- 36
Critical appraisal within sources of evidence	16	If done, present data on critical appraisal of included sources of evidence (see item 12).	19- 36
Results of individual sources of evidence	17	For each included source of evidence, present the relevant data that were charted that relate to the review questions and objectives.	19- 36
Synthesis of results	18	Summarize and/or present the charting results as they relate to the review questions and objectives.	19- 36
**DISCUSSION**			
Summary of evidence	19	Summarize the main results (including an overview of concepts, themes, and types of evidence available), link to the review questions and objectives, and consider the relevance to key groups.	10-12
Limitations	20	Discuss the limitations of the scoping review process.	10-12
Conclusions	21	Provide a general interpretation of the results with respect to the review questions and objectives, as well as potential implications and/or next steps.	10-12
**FUNDING**			
Funding	22	Describe sources of funding for the included sources of evidence, as well as sources of funding for the scoping review. Describe the role of the funders of the scoping review.	.12

**Caption:** JBI = Joanna Briggs Institute; PRISMA-ScR = Preferred Reporting Items for Systematic reviews and Meta-Analyses extension for Scoping Reviews.

* Where *sources of evidence* (see second footnote) are compiled from, such as bibliographic databases, social media platforms, and Web sites.

† A more inclusive/heterogeneous term used to account for the different types of evidence or data sources (e.g., quantitative and/or qualitative research, expert opinion, and policy documents) that may be eligible in a scoping review as opposed to only studies. This is not to be confused with *information sources* (see first footnote).

‡ The frameworks by Arksey and O’Malley (6) and Levac and colleagues (7) and the JBI guidance (4, 5) refer to the process of data extraction in a scoping review as data charting*.*

§ The process of systematically examining research evidence to assess its validity, results, and relevance before using it to inform a decision. This term is used for items 12 and 19 instead of "risk of bias" (which is more applicable to systematic reviews of interventions) to include and acknowledge the various sources of evidence that may be used in a scoping review (e.g., quantitative and/or qualitative research, expert opinion, and policy document). Tricco et al.^(23)^.
