# Supplementary material for: The relationship between bullying and stuttering in schoolchildren: scoping review
Source: Codas. 2026 Aug 7;38(5):e20250428. doi: 10.1590/2317-1782/e20250428en (PMC13520639; doi:10.1590/2317-1782/e20250428en)
Supplement: Translation

# Translation: A relação entre o bullying e a gagueira em escolares: revisão de escopo

**DOI:** 10.1590/2317-1782/e20250428pt

**Authors:** Gislaine Aparecida Folha, Gabrielly Lemos Barbosa, Caio Rodrigues Felix, Hadassa Bighi Lima Baptista

**Affiliations:** 1 Faculdade de Medicina de Ribeirão Preto – FMRP, Universidade de São Paulo – USP - Ribeirão Preto (SP), Brasil.; 2 Núcleo de Apoio à Pesquisa em Morfofisiologia do Complexo Craniofacial da USP Universidade de São Paulo – USP - Ribeirão Preto (SP), Brasil.

**Keywords:** Gagueira,Transtorno da Fluência com Início na Infância, Bullying, Distúrbios da Fala, Criança

## Abstract

**Objetivo:**

mapear e sintetizar evidências científicas sobre a relação entre bullying e gagueira em escolares. Serão investigados os objetivos específicos: (1) o impacto da coocorrência de ambos; (2) estratégias de identificação do bullying em crianças que gaguejam; e (3) abordagens para enfrentamento do bullying nessa população.

**Estratégia de pesquisa:**

Foi conduzida uma Scoping Review seguindo metodologia do Instituto Joanna Briggs. As bases de dados PubMed, Scielo, CAPES e Web of Science foram consultadas com descritores específicos.

**Critérios de seleção:**

foram incluídos artigos em inglês ou português com texto completo, sem limite temporal. Excluídos artigos de opinião, cartas ao editor e estudos que não responderam ao objetivo do trabalho.

**Análise dos dados:**

os dados extraídos incluíram autor, título, objetivos, metodologia, resultados e conclusões, culminando em um fluxograma PRISMA-ScR para organização dos achados.

**Resultados:**

os estudos analisados apontam maior prevalência de bullying, ansiedade e baixa autoestima entre crianças e adolescentes que gaguejam, especialmente no ambiente escolar. Relações sociais fragilizadas e falta de conscientização aumentam o risco de exclusão. O fonoaudiólogo tem papel essencial na orientação e prevenção, embora ainda haja necessidade de pesquisas mais robustas sobre intervenções eficazes.

**Conclusões:**

Este estudo confirmou a relação entre a gagueira e a vivência do bullying, evidenciando impactos na saúde mental e na socialização das pessoas que gaguejam. Além disso, embora escassas, foram reunidas as principais estratégias de intervenção identificadas até o momento, como programas educativos e atividades de prevenção, ainda no ambiente escolar e em conjunto com o corpo acadêmico e familiares.

## INTRODUÇÃO

Embora existam várias definições na literatura, o *bullying* é entendido como atos repetitivos realizados por uma pessoa ou grupo com a intenção de prejudicar um indivíduo ou um grupo que compartilha características comuns, por isso, deve ser considerado inaceitável e inadequado^(1)^. As ocorrências de *bullying* podem se manifestar de forma física, verbal, social ou por meio de interações interpessoais, como o isolamento social^(1)^. Esses comportamentos podem se dar em diversos contextos, como na escola, no ambiente familiar, em interações virtuais (*cyberbullying*) ou entre amigos, existindo um desequilíbrio de poder entre quem pratica o *bullying* e a vítima, que se sente inferior^(1)^.

O *bullying* é considerado como uma epidemia global entre escolares, com taxas que variam entre 49% e 58% dos alunos do ensino fundamental e pode ser um problema significativo para crianças que gaguejam^(1)^ por enfrentarem o *bullying* com maior frequência em relação aos pares que não gaguejam^(2)^. Nas crianças que gaguejam a incidência de *bullying* chega a 81%^(3)^.

O comportamento de *bullying* entre crianças que gaguejam está frequentemente associado a apelidos, difamação, sentimentos de culpa, agressão física e ridicularização^(4)^. No ambiente escolar, isso pode diminuir a autoestima da criança, prejudicar seu desempenho acadêmico, aumentar a rejeição social e levar à depressão, sentimentos de desamparo e solidão. Para crianças com transtornos de fluência da fala, o *bullying* pode exacerbar a gagueira, intensificar emoções negativas e reduzir o progresso terapêutico^(5)^. As consequências do *bullying* podem ser duradouras, afetando até a vida adulta, resultando em níveis elevados de ansiedade social, medo de avaliações negativas e impactos negativos na qualidade de vida^(6,7)^.

Em crianças que gaguejam o *bullying* pode ocorrer porque as disfluências da criança são de difícil compreensão para os outros, que podem não entender por que a fala desta se diferencia do que a sociedade entende como “padrão”. Isso destaca a importância de educar colegas, familiares e educadores sobre a gagueira, para que possam responder de forma construtiva em situações de intimidação^(1)^, especialmente de crianças que muitas vezes não sabem ainda como responder a este tipo de agressão.

Os fonoaudiólogos, em parceria com professores, devem liderar esforços para ajudar crianças que gaguejam a lidar com a intimidação. O empoderamento dessas crianças pode ser uma estratégia eficaz no combate ao *bullying*. No entanto, a falta de entendimento sobre o papel dos fonoaudiólogos nesse aspecto do tratamento ou incertezas sobre como abordar o *bullying* pode dificultar essa atuação^(8)^. Embora os fonoaudiólogos sejam uma referência para essas queixas, nem sempre estão preparados para lidar com esta questão^(1)^.

Com o intuito de organizar as informações e destacar a contribuição do fonoaudiólogo na área, é necessário promover uma melhor compreensão sobre o como *bullying* acontece em escolares, sobre o impacto do *bullying* no Transtorno da Fluência com Início na Infância (“gagueira”), mapear e sintetizar evidências científicas sobre a relação entre *bullying* e gagueira.

Ressalta-se que em uma busca preliminar não foram encontradas na literatura nenhum tipo de estudo de revisão de literatura sobre o tema, evidenciando a importância do presente estudo para a contextualização atual da problemática e levantamento de recomendações para futuras pesquisas centradas na correlação entre o impacto do Transtorno da Fluência com Início na Infância e o *bullying*. Assim, ressalta-se a necessidade de preencher as lacunas na literatura científica a fim de impulsionar estratégias para o enfrentamento desse impasse.

Ao término deste projeto, espera-se evidenciar por meio da elaboração de um fluxograma (PRISMA-ScR*)^(9)^ os dados extraídos da análise bibliográfica sobre a relação entre o *bullying* e a gagueira em escolares.

Assim, os objetivos gerais deste trabalho foram mapear e sintetizar as evidências científicas sobre a relação entre o *bullying* e a gagueira em escolares, destacando os objetivos específicos de: descrever o impacto da coocorrência de ambos, as estratégias de identificação, e as possíveis estratégias de enfrentamento.

## MÉTODOS

### Estratégia de pesquisa

O levantamento da literatura foi realizado por meio de acesso às bases de dados PubMed, Scielo, CAPES e Web of Science, estas bases foram selecionadas devido aos seus critérios metodológicos, como abrangência do tema, qualidade da indexação e relevância temática. A revisão abrangeu publicações até a data de junho de 2025 sem limite temporal anterior.

Foram utilizados os descritores “*Bullying*", "Gagueira", "Taquifemia", "Distúrbios da fluência da fala", "Distúrbios do neurodesenvolvimento da fluência da fala", bem como suas respectivas correspondências em língua inglesa, "*Stuttering*", “*Stutter*” "*Tachyphemia*", "*Fluency disorder*", "*Neurodevelopmental speech fluency disorder*".

Três revisores (GLB, CRF e HBLB) examinaram independentemente os títulos e resumos e determinaram se os estudos atendiam aos critérios de elegibilidade. O treinamento para a seleção pelos revisores das bibliografias foi realizado pela pesquisadora responsável com experiência na área, para que houvesse uniformidade na seleção e coleta dos dados. No Quadro 1 são apresentadas as bases de dados consultadas com os respectivos descritores consultados. Cada revisor alimentou uma planilha do *Software* Excel (*Microsoft® Corporation*) com os títulos, palavras-chave e resumos, pontuando a inclusão ou não de cada trabalho.

**Quadro 1:** Bases eletrônicas e estratégias de busca

**Base eletrônica**	**Estratégia (descritores)**
Pubmed	(Bullying AND Gagueira), (Bullying AND stuttering OR stutter), (Bullying AND Taquifemia), (Bullying AND Tachyphemia), (Bullying AND Distúrbios da fluência da fala), (Bullying AND Fluency disorder), (Bullying AND Distúrbios do neurodesenvolvimento da fluência da fala), (Bullying AND Neurodevelopmental speech fluency disorder)
Scielo	(Bullying AND Gagueira), (Bullying AND stuttering OR stutter), (Bullying AND Taquifemia), (Bullying AND Tachyphemia), (Bullying AND Distúrbios da fluência da fala), (Bullying AND Fluency disorder), (Bullying AND Distúrbios do neurodesenvolvimento da fluência da fala), (Bullying AND Neurodevelopmental speech fluency disorder)
CAPES	(Bullying AND Gagueira), (Bullying AND stuttering OR stutter), (Bullying AND Taquifemia), (Bullying AND Tachyphemia), (Bullying AND Distúrbios da fluência da fala), (Bullying AND Fluency disorder), (Bullying AND Distúrbios do neurodesenvolvimento da fluência da fala), (Bullying AND Neurodevelopmental speech fluency disorder)
Web of Science	(Bullying AND Gagueira), (Bullying AND stuttering OR stutter), (Bullying AND Taquifemia), (Bullying AND Tachyphemia), (Bullying AND Distúrbios da fluência da fala), (Bullying AND Fluency disorder), (Bullying AND Distúrbios do neurodesenvolvimento da fluência da fala), (Bullying AND Neurodevelopmental speech fluency disorder)
Google acadêmico	(Bullying AND Gagueira), (Bullying AND stuttering OR stutter), (Bullying AND Taquifemia), (Bullying AND Tachyphemia), (Bullying AND Distúrbios da fluência da fala), (Bullying AND Fluency disorder), (Bullying AND Distúrbios do neurodesenvolvimento da fluência da fala), (Bullying AND Neurodevelopmental speech fluency disorder)

Após esta seleção, os dois pesquisadores realizaram reuniões de consenso para a manutenção ou não de cada trabalho encontrado para as próximas etapas. Em caso de divergência houve a análise de um terceiro revisor.

### Critérios de elegibilidade

Foram incluídos no estudo os trabalhos que atenderam aos seguintes critérios: artigos com texto completo em línguas inglesa ou portuguesa que contemplaram a busca com os descritores supracitados. Foram excluídos artigos de opinião, cartas ao editor e estudos que não responderam ao objetivo do trabalho. Não foram estabelecidos limites de tempo de publicação.

Apenas os artigos que preencheram todos os critérios de elegibilidade, foram considerados para as análises a seguir.

### Análise dos dados

Nesta etapa os avaliadores foram uma Fonoaudióloga com Doutorado como maior nível de escolaridade e uma graduanda em Fonoaudiologia, para garantir o alinhamento conceitual quanto à temática estudada. Os estudos foram lidos integralmente e julgados pelos avaliadores de maneira independente, de modo que permanecessem apenas trabalhos que contemplassem os objetivos da presente pesquisa, isto é, o impacto da coocorrência do *bullying* e da gagueira, e/ou as estratégias de identificação do *bullying* na gagueira e/ou as estratégias de enfrentamento do *bullying* na gagueira. Nesta etapa, caso novas divergências surgissem, uma reunião de consenso, incluindo um terceiro avaliador, poderia ser solicitada.

Os estudos incluídos na revisão foram registrados em uma planilha com base no protocolo de análise criado para a presente revisão e organizados na plataforma Mendeley. Assim, os dados registrados para a revisão foram: autor, título, ano, idioma, objetivos do trabalho, tipo de trabalho, metodologia, resultados obtidos e conclusões.

O resumo das etapas do processo de seleção dos artigos presentes nesta revisão está disposto no fluxograma da Figura 1. O Fluxograma apresenta a quantidade de estudos encontrados com os descritores supracitados, bem como os motivos das exclusões em cada etapa.

**Figura 1:** Fluxograma do processo de seleção dos estudos

Esta revisão da literatura foi realizada seguindo a metodologia de *Scoping Review proposta pelo Institute Joanna Briggs*- JBI^(9)^. Essa abordagem oferece um processo rigoroso e transparente, sendo comumente utilizada no campo da saúde. Os objetivos principais incluem mapear e resumir evidências, explorar a abrangência da literatura e guiar futuras investigações sobre um conceito central em um determinado domínio de pesquisa. Para isto, a pergunta norteadora da pesquisa deve responder a três perguntas chave, qual a P= População (P), o Conceito (C) e o Contexto (C) da pesquisa (PCC). A clareza da pergunta da revisão auxilia no desenvolvimento do protocolo, pesquisa bibliográfica e revisão de escopo. Assim, a pergunta norteadora deste trabalho foi “Qual a relação entre *bullying* e gagueira em crianças em idade escolar?”, com P: crianças escolares, C: Gagueira e *bullying* e C: período escolar.

O trabalho seguiu rigorosamente o Checklist PRISMA-ScR, como apresentado no Apêndice 1.

## RESULTADOS

### Resultados nas bases eletrônicas de dados

A busca inicial resultou em um levantamento de 119 artigos. Após a exclusão dos artigos duplicados, obteve-se o total de 41 artigos submetidos à primeira etapa de análise. Dos 41 títulos e resumos de artigos, 27 foram selecionados para leitura na íntegra. Destes, apenas 17 corresponderam aos critérios e à pergunta norteadora, estes foram incluídos no estudo. O fluxograma apresentado na Figura 1 demonstra o processo de seleção dos estudos para esta revisão juntamente com o Quadro 2.

**Quadro 2:** Apresentação dos estudos que atenderam aos critérios de elegibilidade

**Autores**	**Título**	**Ano**	**Língua**	**Objetivos do trabalho**	**Tipo de trabalho**	**Metodologia**	**Resultados**	**Conclusões**
Mallick R, Kathard H, Borhan ASM, Pillay M, Thabane L	A cluster randomised trial of a classroom communication resource program to change peer attitudes towards children who stutter among grade 7 students	2018	Inglês	O objetivo principal deste estudo foi determinar o efeito do tratamento seis meses após a intervenção dos participantes da 7ª série (intervenção com Recursos de Comunicação em Sala de Aula [CCR] versus sem CCR) usando pontuações globais da Medida de Resultados de Recursos para Gagueira (SROM) em grupos escolares. O objetivo secundário foi determinar o efeito do tratamento dos participantes da 7ª série nas subescalas da SROM, incluindo Distância Social Positiva (PSD), Pressão Social (SP) e Interação Verbal (VI). O objetivo do subgrupo foi determinar qualquer diferença no resultado primário entre escolas entre e entre grupos quintis (mais baixos e mais altos).	experimental	Depois que as escolas foram estratificadas em subgrupos de quintil inferior e superior (definidos de acordo com a localização geográfica, mensalidade por escola e recursos), elas foram designadas aleatoriamente para grupos de controle e intervenção, compostos por participantes da 7ª série com idade geralmente igual ou superior a 11 anos. Os professores receberam 1 hora de treinamento antes de administrar a intervenção CCR de dose única durante uma sessão de 60 a 90 minutos. A intervenção CCR incluiu uma história social, dramatização e discussão. Todos os participantes assistiram a um vídeo de um CWS e a gagueira foi definida na linha de base. O SROM mediu as atitudes dos colegas seis meses após a intervenção. A randomização foi estratificada por grupo quintil usando uma proporção de alocação de 1:1 . Não foi possível realizar um ensaio cego completo; no entanto, o avaliador dos resultados foi parcialmente cego e o analista também foi cego. Foram utilizadas equações de estimativa generalizadas (GEE), assumindo uma estrutura de correlação permutável para analisar os dados, adotando um princípio de intenção de tratar. A imputação múltipla foi utilizada para lidar com dados em falta. O critério para significância estatística foi definido em alfa = 0,05.	Dez escolas foram aleatoriamente distribuídas entre os grupos controle (k = 5) e intervenção (k = 5), com n = 223 participantes distribuídos entre os grupos intervenção e n = 231 entre os grupos controle. Um total de 454 participantes completou os SROMs nos grupos controle (n = 231) e intervenção (n = 223) e foram analisados no início do estudo e seis meses após a intervenção. Não houve diferença estatisticamente significativa na pontuação global do SROM (diferença média − 0,11; intervalo de confiança de 95% [IC] − 1,56–1,34; p = 0,88). Também não houve diferenças significativas nas subescalas SROM: PSD (diferença média 1,04; IC 95% − 1,02–311; p = 0,32), SP (diferença média − 0,45; IC 95% − 1,22–0,26; p = 0,21) e VI (diferença média 0,05; IC 95% − 1,01–1,11; p = 0,93). Além disso, não houve efeito significativo do subgrupo na pontuação global do SROM (subgrupos do quintil inferior versus superior) (valor p da interação = 0,52). Não foram observados ou relatados danos.	Não foram observadas diferenças estatisticamente significativas. É possível que o período de tempo tenha sido muito curto para observar mudanças nas atitudes dos colegas e que sejam necessários mais estudos para confirmar os resultados deste estudo.
Mallick RB, Thabane L, Borhan ASM, Kathard H	A pilot study to determine the feasibility of a cluster randomised controlled trial of an intervention to change peer attitudes towards children who stutter	2018	Inglês	Este estudo piloto, portanto, teve como objetivo determinar a viabilidade de um ECR explorando: (1) questões processuais e (2) efeito do tratamento do Recurso de Comunicação em Sala de Aula (RCC), uma intervenção para mudar as atitudes dos colegas em relação às crianças que gaguejam.	experimental	Um projeto piloto de RCT estratificado de cluster foi empregado, no qual o recrutamento ocorreu primeiro no nível escolar e depois no nível individual. A taxa de abandono foi relatada na linha de base, 1 e 6 meses após a intervenção. Para o efeito do tratamento, as escolas foram a unidade de randomização e foram randomizadas para receber a intervenção CCR administrada por professores ou a prática usual, usando uma proporção de alocação de 1:1. A medida de resultados de recursos de gagueira (SROM) mediu o efeito do tratamento na linha de base, 1 e 6 meses após a intervenção geral e dentro dos construtos (distância social positiva, pressão social e interação verbal).	Para o recrutamento escolar, 11 escolas foram convidadas a participar e 82% ( n = 9) foram recrutadas. Com base no recrutamento escolar, N = 610 participantes foram elegíveis para este estudo, enquanto apenas n = 449 foram recrutados, onde havia n = 183 no grupo de intervenção e n = 266 no grupo de controle. A taxa de abandono do recrutamento para a linha de base foi a seguinte: intervenção, 23% ( n = 34) e controle, 6% ( n = 15). Em 1 mês, uma taxa de abandono de 7% ( n = 10) foi observada na intervenção e 6% ( n = 15) no grupo de controle, enquanto em 6 meses, taxas de abandono de 7% ( n = 10) e 17% ( n = 44) foram encontradas nos grupos de intervenção e controle, respectivamente. Para o efeito do tratamento no SROM, as diferenças médias estimadas entre os grupos de intervenção e controle foram (intervalo de confiança de 95% (IC): -1,07, 5,11) em 1 mês e 3,01 (IC de 95%: -0,69, 6,69) em 6 meses. Uma diferença estatisticamente significativa foi observada em 6 meses na subescala VI do SROM, com 1,35 (IC de 95%: 0,58, 2,13).	Uma alta taxa de recrutamento de escolas e participantes foi observada com uma alta taxa de abandono de participantes. Diferenças significativas foram notadas apenas 6 meses após a intervenção dentro de um dos construtos do SROM. Essas descobertas sugerem que um futuro estudo RCT é garantido e viável.
Mandrá PP, Cassini M, Gonçalves TC, Kuroishi RCS	Ações educativas em saúde: satisfação em relação à campanha de atenção à gagueira	2017	Português	Investigar a satisfação com as ações educativas em saúde realizadas na Campanha de atenção à Gagueira.	Observacional transversal	A amostra foi composta de documentos institucionais arquivados referentes aos 135 questionários aplicados e totalmente preenchidos na Campanha de Atenção à Gagueira: do tipo diretivo, constituído por questões fechadas, com resposta escalar do tipo Likert para o grau de satisfação, duas dicotômicas sobre o Bullying (“sim”/”não”) e uma aberta sobre “o que faltou na campanha”. Os dados obtidos foram categoriza- dos e tabulados para análise por meio de estatística não paramétrica. Utilizou-se o cálculo das frequências absoluta e relativa das respostas. Para a análise foram divididos em dois grupos de acordo com o local da campanha: G1 (serviço especializado, n=31) e G2 (escolas, n=104).	As ações educativas foram realizadas predominantemente por meio de palestra (89,6%), e de roda de conversa (10,4%). Todos os participantes tiveram acesso ao folheto informativo e à camiseta; 84,4% tiveram acesso ao filme e ao cartaz. Houve satisfação da maioria dos participantes quanto ao tema e período de duração. As explicações foram suficientes para entender o que é bullying (97,9%); 57,6% responderam conhecer alguém que sofreu algum tipo de intimidação (6,0% referente à gagueira). Quanto ao que faltou na campanha, verificou-se ma diversidade de respostas, com maior percentual para “formas de tratamento” (11,9%), seguido por “espancamento nas escolinhas” (0,9%).	Conclusão: Houve satisfação com as ações educativas em saúde na Campanha de Atenção à Gagueira de 2010, quanto ao tema, período de duração, tipo de atividades e materiais acessados.
Blood GW, Boyle MP, Blood IM, Nalesnik GR	Bullying em crianças que gaguejam: percepções e estratégias de intervenção de fonoaudiólogos	2010	Inglês	O estudo atual teve três objetivos principais: (1) determinar a gravidade percebida e a probabilidade de intervenção em episódios de bullying físico, verbal e relacional em alunos que gaguejam por fonoaudiólogos da escola, (2) explorar as percepções dos fonoaudiólogos sobre possíveis estratégias/intervenções que eles achavam que seriam apropriadas com base em seis vinhetas diferentes descrevendo o bullying físico, verbal e relacional de alunos que gaguejam, e (3) determinar quaisquer associações entre as respostas dos fonoaudiólogos e (a) sua idade, (b) o número de anos em que trabalhavam como fonoaudiólogos, (c) o número de crianças que gaguejam atualmente em seus casos e (d) o tamanho do caso, pois podem estar relacionados às percepções dos fonoaudiólogos e suas classificações de estratégias específicas para intervenção.	observacional longitudinal	Para esta pesquisa foi utilizada uma técnica de amostragem aleatória, proporcional, estratificada e probabilística ( Dillman e outros, 2008 , Fink, 2008 ). A amostra foi proporcional ao número de SLPs em cada estado e estratificada para todos os 50 estados. A metodologia de pesquisa por correio foi empregada porque permitiu a maior variedade de participantes potenciais em vários locais nos Estados Unidos. Além disso, é o método preferido para estudar tópicos sensíveis discretamente de forma confidencial e anônima ( Babbie, 2006 , Babbie, 2007 , Dillman e outros, 2008 , Fink, 2008 ).	As principais descobertas deste estudo são que: (1) tanto a seriedade percebida quanto a probabilidade de intervenção foram maiores para o bullying físico, seguido pelo bullying verbal, com o bullying relacional consistentemente classificado como o mais baixo pelos fonoaudiólogos escolares, (2) as referências à gagueira nos cenários de bullying geralmente não foram vistas como mais sérias ou necessitando de intervenção do que os cenários de bullying nos quais a gagueira não foi referenciada. Além disso, a referência à gagueira provocou diferenças significativas nas classificações para apenas duas das nove estratégias de intervenção, e (3) a seriedade percebida, a probabilidade de intervenção e o uso de estratégias de intervenção não diferiram em função de fatores demográficos ou práticas de trabalho.	As conclusões do artigo destacam a necessidade de uma abordagem mais sistemática para abordar o bullying em crianças que gaguejam dentro das práticas de fonoaudiologia. Os fonoaudiólogos reconhecem a importância de integrar estratégias de intervenção que não apenas foquem na fluência da fala, mas também abordem os aspectos sociais e emocionais da gagueira.
Kira CF, Alpes MF, Mandrá PP	Construção e validação de conteúdo de material educativo sobre gagueira	2023	Português	O objetivo foi construir e validar o conteúdo de material ilustrativo para aconselhar familiares e educadores de crianças com gagueira	revisão	Estudo com abordagem metodológica, seguindo as etapas preconizadas em literatura para a validação de material educativo.Foram selecionados três grupos de juízes: oito fonoaudiólogos, 10 educadores de educação infantil e 10 familiares de crianças e adolescentes gagos. Os juízes responderam um questionário autoaplicável para julgar o conteúdo, a linguagem, as ilustrações, o layout e a motivação dos materiais. E, após a leitura do folder deveriam assinalar um dos níveis de concordância (escala Likert): (1) discordo totalmente, (2) discordo, (3) não discordo nem concordo, (4) concordo, (5) concordo totalmente, e depois três questões dicotômicas (sim/não) associadas a um espaço para comentário/sugestões	Foram consideradas sugestões e comentários individuais dos juízes envolvendo conteúdo e linguagem. A versão final em folha A4, modo paisagem e com três abas, disposto da seguinte forma:capa, contracapa com a incidência e tipos (desenvolvimental, neurogênica e psicogênica), diagnóstico e tratamento, atitudes favoráveis e um lembrete sobre o bullying na gagueira, além da definição e principais manifestações da gagueira	O material foi construído e seu conteúdo validado, apresentando alto índice de aceitação e concordância entre os juízes, sendo um suporte para os profissionais da área de saúde e da educação e aos pais acerca da temática da gagueira.
Lowe R, Jelčić Jakšić S, Onslow M, O’Brian S, Vanryckeghem M, Millard S, et al.	Contemporary issues with stuttering: The Fourth Croatia Stuttering Symposium	2021	Inglês	Têm como principais objetivos documentar e discutir questões clínicas atuais relacionadas à gagueira, conforme abordadas durante o simpósio.	revisão	Consiste na documentação das discussões que ocorreram durante o simpósio realizado em 2019. Os autores prepararam um registro escrito que reproduz fielmente os tópicos abordados, com o objetivo de identificar as principais questões clínicas contemporâneas relacionadas à gagueira. A abordagem incluiu a coleta de insights de fonoaudiólogos, acadêmicos e pesquisadores de 29 países, que compartilharam suas experiências e perspectivas sobre o manejo da gagueira.	Indicam que a intervenção precoce é crucial para o manejo eficaz da gagueira, mas ainda não existe consenso sobre a melhor abordagem para sua implementação. O estudo também destaca a dificuldade na tradução de práticas baseadas em evidências para o atendimento clínico e menciona a ansiedade que crianças podem sentir durante o tratamento. Além disso, os autores recomendam a realização de avaliações adequadas para gagueira persistente e sugerem que fonoaudiólogos são os profissionais ideais para fornecer terapia cognitivo-comportamental para os pacientes	Ressaltam a importância da intervenção precoce para o tratamento eficaz da gagueira, embora ainda não haja consenso sobre o timing e a gestão dessas intervenções.
Murphy WP, Yaruss JS, Quesal RW	Enhancing treatment for school-age children who stutter II. Reducing bullying through role-playing and self-disclosure	2007	Inglês	Investigar maneiras de reduzir o bullying sofrido por crianças em idade escolar que gaguejam, utilizando técnicas de role-playing (encenação) e auto-revelação (self-disclosure). O estudo busca avaliar como essas abordagens podem melhorar a comunicação das crianças, aumentar sua confiança e ajudar a lidar de forma eficaz com o bullying no ambiente escolar.	experimental	Crianças em idade escolar que gaguejam participam de sessões de intervenção que utilizam role-playing (encenação) e auto-revelação (self-disclosure). Durante as sessões, as crianças praticam diferentes cenários sociais, simulando situações de bullying, para aprender a responder de maneira eficaz. A auto-revelação também é incentivada, ajudando as crianças a falar abertamente sobre sua gagueira. O impacto dessas intervenções é avaliado com base em mudanças no comportamento das crianças e na redução do bullying relatado.	Os resultados do estudo mostram que as técnicas de role-playing e auto-revelação ajudaram a reduzir o bullying em crianças que gaguejam. As crianças que participaram das intervenções relataram maior confiança ao lidar com situações sociais difíceis e apresentaram melhorias na maneira de abordar o bullying. Houve também uma diminuição significativa nas ocorrências de bullying relatadas após a implementação das técnicas, indicando que essas estratégias foram eficazes para promover respostas mais assertivas e melhorar as interações sociais das crianças.	As técnicas de role-playing e auto-revelação são eficazes na redução do bullying sofrido por crianças que gaguejam. O estudo demonstra que essas intervenções aumentam a confiança das crianças, melhoram suas habilidades de comunicação e as ajudam a lidar com situações de bullying de maneira mais assertiva.
Kikuchi Y, Umezaki T, Sawatsubashi M, Taura M, Yamaguchi Y, Murakami D, Nakagawa T	Experiences of Teasing and Bullying in Children Who Stutter	2019	Inglês	Investigar a frequência e o impacto de experiências de provocação e bullying vividas por crianças que gaguejam, especialmente considerando a idade e o tipo de experiência relatada.	observacional transversal	A amostra contou com 120 crianças que gaguejam (CWS), com idades entre 3 e 12 anos, atendidas em um hospital universitário no Japão, no qual foram entrevistadas individualmente.	66,6% das crianças relataram ao menos uma experiência negativa relacionada à sua fala; As experiências relatadas aumentaram com a idade, especialmente a partir dos 5 anos; A maioria das crianças que passaram por essas situações relatou sentimentos negativos, como tristeza e desconforto.	Muitas crianças que gaguejam são alvo de perguntas, imitações e risos por parte de outras pessoas, mesmo em idades muito precoces. Essas situações são percebidas como desconfortáveis ou tristes pela maioria das crianças. Os autores recomendam que profissionais de saúde perguntem diretamente às crianças sobre essas vivências e intervenham precocemente, promovendo a prevenção do bullying e protegendo a saúde emocional dessas crianças.
Zamprogno MP, Mandrá PP, Gonçalves TC, Jorge TM	Experiências de violência na escola na percepção de pacientes com gagueira	2020	Português	Caracterizar os pacientes com gagueira persistente em idade escolar, quanto às experiências de violência no contexto escolar, autorrelatadas pelos mesmos.	observacional transversal	A amostra foi constituída por 10 pacientes com gagueira persistente em atendimento em um ambulatório de fluência do município de Ribeirão Preto, São Paulo, Brasil, entre 10 e 17 anos, independentemente do sexo e das características da gagueira. O instrumento de coleta foi um questionário autoaplicável contendo 11 questões de múltipla escolha. Os dados foram analisados descritivamente, por apresentação da frequência de respostas.	Quase metade dos pacientes com gagueira afirmaram sofrer bullying, por meio do recebimento de apelidos, culpabilização por tudo o que acontece, difamações, ataques físicos e zombarias. A sala de aula foi o local mais mencionado para a ocorrência do bullying. Como reações à violência sofrida, foram mencionados: ‘falar com os amigos, professores/diretores e familiares’, além de ‘tristeza’ e‘vontade de mudar de escola’.	Apesar do reduzido número amostral, foi possível notar dados alarmantes e a importância de ações educativo-preventivas no ambiente escolar, tanto para a temática do “bullying”, como da “gagueira”.
Bernard RFL, Norbury CF.	Factors Associated With Symptoms of Anxiety and Depression in Children Who Stutter	2023	Inglês	Este estudo considera a associação entre sintomas de ansiedade e depressão e gagueira, bem como fatores infantis, familiares e contextuais que podem afetar essa associação.	observacional transversal	Trinta e cinco crianças em idade escolar que gaguejam completaram a Revised Children's Anxiety and Depression Scale-Short Version. Ajustamos modelos de regressão para examinar a associação entre sintomas de ansiedade e depressão com bullying, gravidade da gagueira, histórico familiar de saúde mental adversa e idade em crianças que gaguejam.	Histórico familiar de saúde mental adversa foi encontrado para prever significativamente pontuações de ansiedade e depressão. A idade também previu pontuações de depressão, com crianças mais velhas relatando pontuações mais altas.	O histórico familiar de saúde mental adversa está associado a maiores sintomas internalizantes auto relatados em crianças que gaguejam. A interação entre criança, família e fatores contextuais pode mudar com a idade, e isso requer maior exploração em estudos longitudinais maiores. A associação entre bullying e escores de ansiedade indica a importância de iniciativas antibullying na promoção do desenvolvimento psicossocial em crianças em idade escolar que gaguejam. Este estudo também destaca a contribuição de fatores de risco conhecidos para a saúde mental, como histórico familiar, para a variabilidade no relato de sintomas.
Silva LK, Martins-Reis V de O, Maciel TM, Ribeiro JKBC, Souza MA de, Chaves FG	Gagueira na escola: efeito de um programa de formação docente em gagueira	2016	Português	Verificar o que docentes de escolas públicas e privadas sabem sobre gagueira, bem como verificar a eficácia do Programa de Formação Docente em Gagueira na ampliação desses conhecimentos.	observacional transversal	Participaram do estudo 137 docentes da educação infantil. Inicialmente os docentes responderam a um questionário sobre gagueira. Em seguida, 75 docentes participaram do Programa de Formação Docente em Gagueira com duração de quatro horas. Um mês após participação no programa, os docentes responderam novamente ao questionário.	Depois da formação, notou-se um aumento no percentual daqueles que consideram baixa a prevalência de pessoas que gaguejam na população. Os entrevistados passaram a relatar que a gagueira é mais frequente no gênero masculino. Houve aumento daqueles que consideram a gagueira hereditária. Diminuiu a incidência de educadores que acreditavam que a gagueira é psicológica. A maioria dos entrevistados passou a acreditar na multicausalidade da gagueira. Diminuiu o índice de educadores que afirmavam que a gagueira é emocional. Houve melhor entendimento de algumas atitudes que os educadores podem ter na tentativa de ajudar uma pessoa que gagueja.	Os educadores possuíam algum conhecimento sobre gagueira, mas insuficiente para diferenciá-la dos demais distúrbios de linguagem. O programa ampliou os conhecimentos em relação à gagueira. Entretanto, mostrou-se mais efetivo para as características da gagueira do que para as atitudes dos educadores.
Davis S, Howell P, Cooke F.	Sociodynamic relationships between children who stutter and their non-stuttering classmates	2002	Inglês	Investigar as interações sociais entre crianças que gaguejam e seus colegas que não gaguejam. O estudo busca entender as dinâmicas de aceitação, rejeição ou isolamento social que podem ocorrer entre esses grupos, bem como o impacto da gagueira nas relações de amizade, comportamento em grupo e na percepção social dos colegas	observacional transversal	A metodologia do artigo consiste em uma análise observacional das interações sociais entre crianças que gaguejam e seus colegas. Os pesquisadores utilizaram questionários e observações diretas em ambiente escolar para avaliar comportamentos sociais, níveis de aceitação, rejeição e redes de amizade entre os participantes. O foco foi identificar padrões de interação e entender como a gagueira influencia as relações dentro do grupo.	Os resultados do artigo mostram que crianças que gaguejam enfrentam maiores níveis de isolamento social e rejeição por parte de seus colegas não-gagos. As interações sociais são mais limitadas, e elas tendem a ter menos amigos. No entanto, alguns colegas demonstram apoio e aceitação, o que varia conforme o ambiente escolar e as dinâmicas do grupo. O estudo destaca a importância de intervenções sociais para melhorar a inclusão dessas crianças	Crianças que gaguejam enfrentam desafios sociais significativos em suas interações com colegas, como exclusão e dificuldades em formar amizades. O estudo sugere que essas dificuldades podem impactar seu desenvolvimento social e emocional. Os autores recomendam que escolas implementem programas de conscientização e intervenções que promovam a inclusão, para melhorar as experiências sociais de crianças que gaguejam.
McAllister J, Skinner J, Hayhow R, Heron J, Wren Y.	The Association Between Atypical Speech Development and Adolescent Self-Harm	2023	Inglês	Os objetivos do estudo são investigar a relação entre o desenvolvimento atípico da fala em crianças e a automutilação na adolescência, além de explorar como dificuldades de comunicação podem impactar a saúde mental dos adolescentes. O estudo também visa destacar a importância de intervenções precoces para crianças com essas dificuldades, a fim de prevenir comportamentos autolesivos no futuro.	observacional transversal	Através da análise de um banco de dados, os pesquisadores coletaram informações sobre características demográficas e fatores de risco, utilizando escalas padronizadas para avaliar a relação entre o desenvolvimento da fala e comportamentos autolesivos.	O estudo encontrou uma associação significativa entre o desenvolvimento atípico da fala em crianças e o aumento do risco de automutilação na adolescência. Os resultados indicam que as crianças com dificuldades de comunicação apresentam maior probabilidade de desenvolver problemas emocionais e comportamentais, incluindo autolesões. Os autores enfatizam a necessidade de intervenções precoces para apoiar crianças com desenvolvimento da fala atípico, a fim de mitigar esses riscos na adolescência.	As conclusões do estudo destacam que o desenvolvimento atípico da fala em crianças está associado a um aumento do risco de automutilação na adolescência
Kara I, Mutlu Aİ, Miraloğlu K	Comparison of Participation in Online Games and Communication Experiences of School-Age Children Who Do and Do not Stutter: Exploratory Study	2023	Inglês	Investigar se há diferenças significativas na participação em jogos online e nas experiências de comunicação entre crianças que gaguejam (CWS) e aquelas que não gaguejam (CWNS). O estudo também busca entender se essas interações no ambiente de jogos podem influenciar as características de fala e a ocorrência de comportamentos como bullying, fornecendo insights importantes para compreender como o ambiente digital afeta a comunicação de crianças que gaguejam	observacional transversal	Aplicação de questionários online para avaliar os hábitos de participação em jogos multiplayer, a frequência de uso e as características de fala durante o jogo. A pesquisa envolveu 91 crianças que gaguejam e 116 que não gaguejam, permitindo comparações nas experiências de comunicação e na ocorrência de comportamentos de bullying durante os jogos	Mostraram que crianças que gaguejam e aquelas que não gaguejam participam de jogos online de maneira semelhante, sem diferenças significativas na frequência ou duração. No entanto, as crianças que gaguejam relataram menos ocorrências de comportamentos de bullying durante os jogos, indicando que o ambiente online pode ser mais seguro e favorável para elas	Ambientes de jogos online podem oferecer oportunidades positivas para a comunicação e um espaço mais seguro para crianças que gaguejam
Karahan Tığrak T, Kulak Kayıkcı ME, Kirazlı MÇ, Tığrak A	Emotional and behavioural problems of children and adolescents who stutter: Comparison with typically developing peers	2020	Inglês	Investigar e comparar os problemas emocionais e comportamentais em crianças e adolescentes que gaguejam com aqueles de seus pares em desenvolvimento típico. O estudo busca identificar diferenças significativas nos níveis de ansiedade, depressão e outras dificuldades emocionais e comportamentais entre os grupos, além de compreender melhor o impacto da gagueira no bem-estar emocional das crianças e adolescentes.	observacional transversal	Consiste na comparação de dois grupos: crianças e adolescentes que gaguejam e crianças em desenvolvimento típico. Os pesquisadores utilizaram questionários para coletar dados sobre problemas emocionais e comportamentais, aplicando instrumentos padronizados, como o Strengths and Difficulties Questionnaire (SDQ), que avaliam aspectos como hiperatividade, dificuldades emocionais e comportamentais. A amostra incluiu crianças e adolescentes de diversas idades, e os dados foram analisados estatisticamente para identificar diferenças significativas entre os grupos.	Crianças e adolescentes que gaguejam apresentam níveis significativamente mais altos de problemas emocionais e comportamentais em comparação com seus pares em desenvolvimento típico. Os dados mostraram que esses indivíduos estavam mais propensos a relatar dificuldades como ansiedade, depressão e comportamentos de hiperatividade. Especificamente, as análises revelaram que os participantes que gaguejam apresentaram maiores escores no Strengths and Difficulties Questionnaire (SDQ) em várias áreas, o que sugere um impacto negativo da gagueira no bem-estar emocional. Esses resultados destacam a necessidade de suporte psicológico e intervenções específicas para crianças que gaguejam, visando melhorar sua saúde mental e social.	Os autores enfatizam a importância de reconhecer essas dificuldades e sugerem que os profissionais de saúde e educação devem implementar intervenções que abordem tanto a gagueira quanto as questões emocionais associadas.
Logan KJ, Mullins MS, Jones KM	The depiction of stuttering in contemporary juvenile fiction: Implications for clinical practice	2008	Inglês	Analisar como a gagueira é representada na literatura juvenil contemporânea e discutir as implicações dessas representações para a prática clínica em fonoaudiologia. Os autores buscaram entender se os livros poderiam ser utilizados como ferramentas educacionais ou terapêuticas no contexto do tratamento da gagueira em crianças e adolescentes	revisão	Selecionar e revisar criticamente 29 livros de ficção juvenil publicados após 1988 que apresentavam personagens crianças ou adolescentes com gagueira. Para a seleção, os autores realizaram buscas em bibliotecas, sites de livrarias, bases acadêmicas e consultaram especialistas e bibliotecários. Os livros selecionados foram avaliados com base na fidelidade às características clínicas da gagueira, profundidade dos temas abordados, fatores pessoais e sociais, e possíveis ganhos funcionais apresentados pelos personagens	A maioria das histórias retrate personagens que inicialmente enfrentam dificuldades significativas relacionadas à gagueira — como isolamento, bullying e baixa autoestima —, muitas delas mostram evolução positiva ao longo da narrativa, ainda que nem sempre associada a tratamento profissional. Em geral, os livros abordam bem os aspectos emocionais e sociais da gagueira, mas frequentemente apresentam explicações imprecisas ou simplificadas quanto às causas e ao tratamento do transtorno, reforçando a ideia de que ele estaria ligado a traumas emocionais ou superado por meio de apoio afetivo e aceitação social.	Os autores apontam que a literatura juvenil pode ser um recurso valioso para uso em intervenções fonoaudiológicas, principalmente em atividades que envolvem educação, promoção de empatia, desmistificação do transtorno e estímulo à autoexpressão. Apesar das limitações — como a pouca menção à terapia fonoaudiológica formal e o excesso de foco em fatores emocionais —, os livros analisados demonstram potencial para auxiliar no trabalho clínico, especialmente quando utilizados com orientação profissional e complementados com informações corretas sobre a gagueira.
Blood GW; Blood IM	Preliminary Study of Self-Reported Experience of Physical Aggression and Bullying of Boys Who Stutter: Relation to Increased Anxiety	2007	Inglês	Investigar a relação entre a experiência de agressão física e bullying em meninos que gaguejam, analisar o impacto dessas experiências na ansiedade dos meninos e identificar padrões de agressão e bullying relatados pelos participantes, buscando compreender como esses fatores afetam a saúde emocional e psicológica deles.	observacional transversal	Foram coletados dados por meio de questionários autoaplicáveis. Os participantes, que eram meninos que gaguejam, relataram suas experiências de agressão física e bullying, além de níveis de ansiedade. A análise dos dados incluiu a comparação das respostas dos meninos em relação às suas experiências de bullying e os níveis de ansiedade relatados, buscando identificar relações significativas entre esses fatores.	Meninos que gaguejam relataram experiências significativas de agressão física e bullying. Esses relatos foram associados a níveis mais altos de ansiedade entre os participantes. O estudo sugere que a gagueira pode aumentar a vulnerabilidade desses meninos a experiências de bullying, o que, por sua vez, contribui para a ansiedade e outros problemas emocionais.	A gagueira pode aumentar a exposição de meninos a agressões físicas e bullying, resultando em níveis elevados de ansiedade. Os autores enfatizam a importância de reconhecer essas experiências e suas repercussões emocionais, sugerindo que intervenções direcionadas são necessárias para oferecer apoio a meninos que gaguejam.

## DISCUSSÃO

Este trabalho buscou mapear e sintetizar evidências científicas sobre a relação entre o *bullying* e a gagueira em escolares levando em consideração o possível impacto, as estratégias de identificação e de enfrentamento quando há a coocorrência de ambos.

Dos 17 estudos selecionados para análise, a maioria indicou alta prevalência de *bullying* ^(4,10-15)^, níveis elevados de ansiedade^(10,15)^ e menor autoestima^(10)^ entre crianças que gaguejam em comparação com as crianças que não gaguejam. Os adolescentes que gaguejam têm um risco significativamente maior de sofrer *bullying* do que aqueles que não gaguejam^(12,16)^. Esses achados reforçam a existência de uma relação significativa entre a ocorrência de bullying e a gagueira, sugerindo que o transtorno do desenvolvimento da fluência da fala na infância, pode atuar tanto como fator de vulnerabilidade quanto como alvo de estigmatização em contextos escolares. Esta afirmação também dá-se pelo fato de que a sala de aula foi apontada como o principal ambiente onde essas situações ocorrem, ressaltando a urgência de estratégias preventivas no ambiente escolar^(4)^.

Diversas pesquisas também relataram que crianças que gaguejam enfrentam rejeição por parte dos colegas, possuem menor *status* social e vivenciam mais dificuldades na formação de amizades^(17)^. A qualidade da relação entre professor e aluno, assim como a conscientização dos adultos, foram identificadas como fatores relevantes que podem influenciar o risco de exposição ao *bullying* ^(18)^. Como estratégia de enfrentamento alguns estudos destacaram que programas educativos sobre gagueira e estratégias de prevenção ao *bullying* nas escolas têm potencial para melhorar a percepção e a atitude dos colegas^(17,19)^.

O papel do fonoaudiólogo mostra-se fundamental nesse contexto, atuando não apenas na identificação como na orientação de crianças sobre a gagueira e o *bullying*, incentivando a aceitação e ensinando formas assertivas de resposta^(12,20)^. No entanto, vale ressaltar que a base de evidências ainda apresenta limitações, devido ao predomínio de delineamentos transversais dos trabalhos encontrados, ao uso de instrumentos variados de avaliação e à presença de dados incompletos, o que pode comprometer a generalização e a robustez das conclusões aqui apontadas.

Embora no Brasil a Fonoaudiologia conte com uma especialidade em Fluência conforme disposto na Resolução CFFa nº 507/2017^(21)^, em que compete ao fonoaudiólogo a orientação tanto da família, quanto de outros profissionais para a identificação de transtornos da fluência, bem como conduta adequada frente aos indivíduos com tais alterações, bem como a realização de estudos visando o desenvolvimento e aprofundamento dos conhecimentos técnicos e científicos no que concerne a área da fluência de fala, nesta presente revisão, apenas quatro^(4,11,19,22)^ dos trabalhos encontrados foram de autores brasileiros.

## CONCLUSÃO

Este estudo confirmou a relação entre a gagueira e a vivência do *bullying*, evidenciando impactos na saúde mental e na socialização das pessoas que gaguejam. Além disso, embora escassas, foram reunidas as principais estratégias de intervenção identificadas até o momento, como programas educativos e atividades de prevenção, ainda no ambiente escolar e em conjunto com o corpo acadêmico e familiares.

A partir disto, futuras investigações poderão avaliar quais intervenções são mais eficazes e como podem ser aplicadas na prática clínica. Ressalta-se a necessidade de novos estudos observacionais e experimentais, com amostras mais amplas, que investiguem o funcionamento e os efeitos das intervenções terapêuticas no cotidiano.

## Apêndice 1 Checklist PRISMA-ScR

Preferred Reporting Items for Systematic reviews and Meta-Analyses extension for Scoping Reviews (PRISMA-ScR) Checklist

**Table:**

**SECTION**	**ITEM**	**PRISMA-ScR CHECKLIST ITEM**	**REPORTED ON PAGE #**
**TITLE**			
Title	1	Identify the report as a scoping review.	1
**ABSTRACT**			
Structured summary	2	Provide a structured summary that includes (as applicable): background, objectives, eligibility criteria, sources of evidence, charting methods, results, and conclusions that relate to the review questions and objectives.	1
**INTRODUCTION**			
Rationale	3	Describe the rationale for the review in the context of what is already known. Explain why the review questions/objectives lend themselves to a scoping review approach.	5
Objectives	4	Provide an explicit statement of the questions and objectives being addressed with reference to their key elements (e.g., population or participants, concepts, and context) or other relevant key elements used to conceptualize the review questions and/or objectives.	6
**METHODS**			
Protocol and registration	5	Indicate whether a review protocol exists; state if and where it can be accessed (e.g., a Web address); and if available, provide registration information, including the registration number.	8
Eligibility criteria	6	Specify characteristics of the sources of evidence used as eligibility criteria (e.g., years considered, language, and publication status), and provide a rationale.	7
Information sources*	7	Describe all information sources in the search (e.g., databases with dates of coverage and contact with authors to identify additional sources), as well as the date the most recent search was executed.	6
Search	8	Present the full electronic search strategy for at least 1 database, including any limits used, such that it could be repeated.	6
Selection of sources of evidence†	9	State the process for selecting sources of evidence (i.e., screening and eligibility) included in the scoping review.	7
Data charting process‡	10	Describe the methods of charting data from the included sources of evidence (e.g., calibrated forms or forms that have been tested by the team before their use, and whether data charting was done independently or in duplicate) and any processes for obtaining and confirming data from investigators.	8
Data items	11	List and define all variables for which data were sought and any assumptions and simplifications made.	7
Critical appraisal of individual sources of evidence§	12	If done, provide a rationale for conducting a critical appraisal of included sources of evidence; describe the methods used and how this information was used in any data synthesis (if appropriate).	6
Synthesis of results	13	Describe the methods of handling and summarizing the data that were charted.	9
**RESULTS**			
Selection of sources of evidence	14	Give numbers of sources of evidence screened, assessed for eligibility, and included in the review, with reasons for exclusions at each stage, ideally using a flow diagram.	8 and 38
Characteristics of sources of evidence	15	For each source of evidence, present characteristics for which data were charted and provide the citations.	19- 36
Critical appraisal within sources of evidence	16	If done, present data on critical appraisal of included sources of evidence (see item 12).	19- 36
Results of individual sources of evidence	17	For each included source of evidence, present the relevant data that were charted that relate to the review questions and objectives.	19- 36
Synthesis of results	18	Summarize and/or present the charting results as they relate to the review questions and objectives.	19- 36
**DISCUSSION**			
Summary of evidence	19	Summarize the main results (including an overview of concepts, themes, and types of evidence available), link to the review questions and objectives, and consider the relevance to key groups.	10-12
Limitations	20	Discuss the limitations of the scoping review process.	10-12
Conclusions	21	Provide a general interpretation of the results with respect to the review questions and objectives, as well as potential implications and/or next steps.	10-12
**FUNDING**			
Funding	22	Describe sources of funding for the included sources of evidence, as well as sources of funding for the scoping review. Describe the role of the funders of the scoping review.	.12

**Legenda:** JBI = Joanna Briggs Institute; PRISMA-ScR = Preferred Reporting Items for Systematic reviews and Meta-Analyses extension for Scoping Reviews.

* Where *sources of evidence* (see second footnote) are compiled from, such as bibliographic databases, social media platforms, and Web sites.

† A more inclusive/heterogeneous term used to account for the different types of evidence or data sources (e.g., quantitative and/or qualitative research, expert opinion, and policy documents) that may be eligible in a scoping review as opposed to only studies. This is not to be confused with *information sources* (see first footnote).

‡ The frameworks by Arksey and O’Malley (6) and Levac and colleagues (7) and the JBI guidance (4, 5) refer to the process of data extraction in a scoping review as data charting*.*

§ The process of systematically examining research evidence to assess its validity, results, and relevance before using it to inform a decision. This term is used for items 12 and 19 instead of "risk of bias" (which is more applicable to systematic reviews of interventions) to include and acknowledge the various sources of evidence that may be used in a scoping review (e.g., quantitative and/or qualitative research, expert opinion, and policy document). Tricco et al.^(23)^
